# Regulation of Limbal Epithelial Stem Cells: Importance of the Niche

**DOI:** 10.3390/ijms222111975

**Published:** 2021-11-05

**Authors:** Sarah Y. T. Robertson, JoAnn S. Roberts, Sophie X. Deng

**Affiliations:** Jules Stein Eye Institute, University of California, Los Angeles, CA 94143, USA; syrobertson@mednet.ucla.edu (S.Y.T.R.); jsroberts@ceils.ucla.edu (J.S.R.)

**Keywords:** cornea, limbal stem cells, signaling, mechanotransduction, extracellular matrix, stem cell niche

## Abstract

Limbal epithelial stem/progenitor cells (LSCs) reside in a niche that contains finely tuned balances of various signaling pathways including Wnt, Notch, BMP, Shh, YAP, and TGFβ. The activation or inhibition of these pathways is frequently dependent on the interactions of LSCs with various niche cell types and extracellular substrates. In addition to receiving molecular signals from growth factors, cytokines, and other soluble molecules, LSCs also respond to their surrounding physical structure via mechanotransduction, interaction with the ECM, and interactions with other cell types. Damage to LSCs or their niche leads to limbal stem cell deficiency (LSCD). The field of LSCD treatment would greatly benefit from an understanding of the molecular regulation of LSCs in vitro and in vivo. This review synthesizes current literature around the niche factors and signaling pathways that influence LSC function. Future development of LSCD therapies should consider all these niche factors to achieve improved long-term restoration of the LSC population.

## 1. Introduction

Light first enters the eye through the cornea, the outermost transparent layer, which is important both as an environmental barrier and in the refraction of light. Proportionally, 65–75% of the angular modifications of light occurs through the cornea, so the cornea is crucial for properly orienting and focusing images onto the retina [1]. The cornea is a complex multilayered tissue with each layer suited for distinct functions [2,3]. The outer layer of the corneal is lined by stratified epithelium that is constantly shed through blinking or rubbing of the eyes. Just as the epidermis of the skin and intestinal lining are renewed through stem cell asymmetric division, migration, and proliferation, the corneal epithelium is renewed by a specific stem cell lineage residing in the limbus, the region lining the circumference of the cornea and adjacent to the sclera [4]. Limbal epithelial stem/progenitor cells (LSCs), which primarily reside in the niche provided by the crypt-like structures including limbal crypts, limbal lacunae, and Palisades of Vogt in human tissue, are quiescent stem cells that can be activated to divide symmetrically and asymmetrically, producing a proliferative progenitor cell that will give rise to mature corneal epithelial cells [5,6]. The homeostasis of the corneal epithelium is modeled using the X-Y-Z hypothesis, which states that LSCs differentiate into transient amplifying cells (TACs), which migrate centripetally to become fully differentiated corneal epithelium, which are then shed from the corneal surface [6]. In the mouse and other mammalian systems, stem/progenitor cells reside in the basal epithelial layer across the whole cornea, while in humans the central cornea lacks a stem/progenitor cell population [7,8,9].

The niche provides not only a protective environment, but also the necessary factors to maintain stem properties of LSCs and initiate differentiation pathways in response to external cues [10]. Epithelial regeneration is carefully regulated through various molecular pathways, which are activated and inhibited through autocrine and paracrine signals, and cues from the extracellular matrix and mechanical stimuli [3,11,12,13,14,15,16,17]. The inability to replace the lost cells could result in the loss of transparency of the cornea, impaired wound healing, and possible loss/deterioration of vision [18,19].

Dysfunction or destruction of the LSCs or their niche results in a pathological condition known as limbal stem cell deficiency (LSCD) [20]. LSCD causes pain, inflammation, and vision loss in patients due to the invasion of conjunctival epithelial cells into the corneal epithelium because of the inability of LSCs to replenish corneal epithelial cells. Treatment of LSCD requires restoration of the LSC population and its niche. The majority of LSCD cases are either unilateral or sub-total bilateral LSCD, as residual LSCs could be found in eyes with clinical features of total LSCD [21]. In these patients, transplantation of autologous LSCs, either by cultivated LSCs or direct tissue transplantation, is a viable and preferred option [22,23]. While LSCs from the patient’s healthy tissue cultivated ex vivo on human amniotic membrane (HAM) is emerging as an effective, donor tissue-free, and xenobiotic-free alternative to traditional surgical treatments of LSCD, the success of an LSC transplant is highly dependent on the percentage and amount of undifferentiated LSCs in culture [24,25,26]. Therefore, it is imperative to understand niche factors involved in the regulation of LSCs in order to improve the outcome of current LSCD treatment as well as develop new therapy.

This review will discuss the current findings on the integration of molecular and mechanical signaling factors that regulate LSC quiescence, self-renewal, differentiation, migration, and proliferation in vivo and in vitro. While recent reviews have been focused on the limbal niche structure [27,28], diseases involving LSC function [29], and advances in LSC bioengineering and LSCD diagnosis [22], here we present an in-depth analysis of molecular regulation and the influence of surrounding physical properties governing the fate of LSCs.

## 2. Signaling Cascades in the LSC Niche and in the Regulation of LSCs

**Figure 1:**
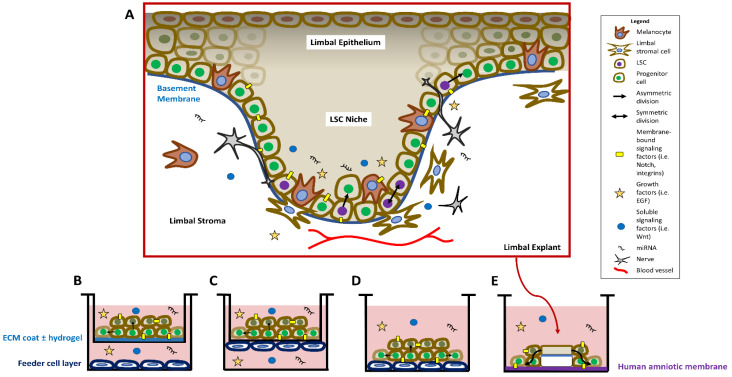
Overview of the structure of the in vivo compared to the in vitro limbal stem cell (LSC) niche. (**A**) In the in vivo niche, LSCs reside primarily in the basal limbal epithelium in the crypts or Palisades of Vogt. Quiescent LSCs (purple nuclei) can be activated to divide asymmetrically into proliferative progenitor cells (green nuclei), or divide symmetrically to maintain the stem cell pool. LSC regulation is maintained by soluble and membrane-bound signaling factors, and microRNAs. The limbal niche harbors melanocytes, nerves, blood vessels, and stromal cells that contribute to the support of the LSCs. (**B**–**E**) Four commonly used methods of in vitro LSC culture. (**B**) LSCs receive structural support from an ECM-and/or hydrogel-coated cell culture insert. Growth factors are provided to the LSCs through a feeder cell layer and the growth medium. (**C**) In the 3D culture model, LSCs and the 3T3 feeder layer are grown on opposite sides of a transwell membrane. (**D**) LSCs are cultivated directly on top of the feeder cell layer, which provides both structural support and growth factors. (**E**) A limbal explant is seeded on a human amniotic membrane, and LSCs proliferate and migrate from the explant.

Cultivated LSCs are a valuable tool for studying LSC regulation and potential LSCD treatments. Methods of cultivating LSCs aim to mimic the in vivo niche conditions by providing ECM and necessary growth factors. A comparison of the in vivo LSC niche and common in vitro methods of maintaining LSCs in culture is shown in Figure 1. LSCs are in contact with the extracellular matrix (ECM) that makes up the basement membrane, mesenchymal cells in the adjacent stroma, and other cell types in the epithelial layer such as melanocytes (Figure 1A). The niche also houses nerves [15,30,31], blood vessels [32,33], and innate immune cells [34,35,36], and the roles that these different cell types play in the niche is a topic of ongoing research. The limbal stroma potentially contains a population of telocytes in the mouse [37], but further research is needed to confirm this population in the human limbus, distinguish these telocytes from other stromal cells, and determine their function in maintaining the human limbal niche. The niche is also comprised of soluble signaling factors, extracellular vesicles, growth factors, and microRNAs that also contribute to the regulation of LSCs. ECM coating such as fibronectin, collagen, or laminin is commonly used in cell culture vessels with or without hydrogels to mimic the structure and properties of the basement membrane (Figure 1B). A layer of feeder cells is often added to provide growth factors to the cultivated LSCs in several configurations: completely separated from the LSCs (Figure 1B), closely adjacent to the LSCs but on the opposite side of a transwell membrane (Figure 1C), or directly co-cultured with the LSCs (Figure 1D). Limbal explants containing LSCs, basement membrane, and adjacent stroma cultivated on HAM (Figure 1E) also preserve cell–cell and cell–matrix interactions for the LSCs. Feeder cells or a surrogate matrix such as HAM are often required in the absence of these niche factors to achieve a good expansion efficiency. Cell culture media components, feeder cells, HAM, and even the LSCs themselves contribute both soluble growth and signaling factors and ECM support to the expanding sheet of LSCs.

While ex vivo cultivated LSCs are a promising treatment for LSCD, removing quiescent LSCs from their native environment to culture places a significant stress on the LSCs. In the absence or dysregulation of the proper molecular signals, growth factors, and/or mechanical cues, LSCs lose their stemness, rendering them unsuitable for transplant unless proper niche factors are provided to preserve their stemness. Therefore, understanding the molecular regulation of LSCs by niche factors is crucial for bioengineering an ex vivo environment for the LSCs that preserves the LSC phenotype in culture. This section of the review synthesizes data on the role of Wnt, TGFβ/BMP, Notch, and Shh pathways in the regulation of LSCs and corneal epithelial differentiation in vitro and in vivo.

### 2.1. Canonical and Non-Canonical Wnt Signaling Improve LSC Proliferation and Maintenance

Wnt signaling influences the ability of stem cells to renew, differentiate, commit to cell fate decisions, and proliferate [38,39,40]. In the absence of Wnt ligands, the transcription factor β-catenin is associated with its destruction complex in the cytoplasm where it is targeted for proteosomal degradation or associated with cadherin–catenin adhesion complexes at the plasma membrane [41,42,43,44]. The binding of secreted Wnt ligands to Frizzled (Fzd) receptors activates three main pathways: the canonical Wnt/β-catenin pathway, the non-canonical Wnt/Ca^2+^ pathway, and the non-canonical Wnt/planar cell polarity pathway. In the canonical pathway, Wnt oligomerization with Fzd and its co-receptor LRP5/6 results in the inactivation of the β-catenin destruction complex, which allows translocation of β-catenin from the cytoplasm to the nucleus, where it activates T-cell factor/lymphoid enhancer factor (TCF/LEF) target genes [45,46].

The non-canonical Wnt pathways are generally considered β-catenin independent because they do not involve β-catenin. The Wnt/Ca^2+^ pathway activates calmodulin kinase II (CamKII) and induces calcium release from the endoplasmic reticulum [47]. A few Wnt ligands have been shown to cause both release of Ca^2+^ into the cytoplasm and nuclear translocation of β-catenin [48]. The Wnt/PCP pathway involves alternate co-receptors, ROR or RYK, and activates c-Jun N-terminal kinase (JNK) and RhoA, which downstream leads to restructuring of the cytoskeleton according to polarity signals [49,50,51].

Many Wnt ligands, receptors, and regulators are differentially expressed in either the cornea or limbus [52,53,54]. The Wnt ligands Wnt2, Wnt6, Wnt11, and Wnt16b are preferentially expressed in the limbus where LSCs reside [53,55]. Inhibitors of canonical Wnt signaling such as WIF1, DKK1, and SFRP5 are also upregulated in the limbus relative to the cornea. The nuclear localization of β-catenin was also mostly detected in basal limbal cells, whereas membrane localization was found on all corneal and limbal epithelial cells [53]. TCF4, a transcription factor that interacts with β-catenin in the nucleus, is also expressed on the basal layer of the limbal epithelium, colocalizing with the stemness markers p63 and ABCG2 [56,57]. MicroRNAs (miRs) such as miR-10b, miR-150-5p, miR-21-5p, miR-1910-5p, miR-10a-5p, and miR-103/107 family are differentially expressed in the basal limbal epithelium and target components of Wnt signaling [58,59,60].

The role of canonical Wnt signaling in LSC maintenance and differentiation has been investigated using explant culture and single LSCs cultivated on a feeder cell layer of mouse 3T3 fibroblasts. Using a single-cell culture system, lithium chloride, an activator of the Wnt canonical pathway, improves proliferation of LSCs and colony-forming efficiency [53]. The use of a Wnt mimic, MFH-ND, was shown to improve the stem cell phenotype in cultivated LSCs [61]. Moreover, if canonical signaling alone is inhibited using the LRP5/6 inhibitor IC15, LSC proliferation is reduced accompanied by a loss of the stem/progenitor cell population [62]. Inhibition of canonical Wnt signaling with XAV939 also increased the percentage of cells expressing the differentiation marker K12 and decreased the colony-forming efficiency of LSCs cultivated on human limbal niche cells [63]. Knockdown of TCF4 using siRNA decreases proliferation and surviving expression in human corneal epithelial cells grown from limbal explants, suggesting that a canonical Wnt/β-catenin/TCF4/4urviving pathway is involved in cultivated LSC proliferation [64]. Similarly, activation of Wnt with the DKK inhibitor IIIC3 also improves the LSC stem cell phenotype. Conversely, high concentrations of IIIC3 decrease LSC colony-forming efficiency and proliferation, and low concentrations of IIIC3 increase the percentage of cells expressing K12 [62]. Possible explanations for these data include (1) IIIC3 may bind to and inhibit LRP5/6 at high concentrations due to structural similarity with DKK, or (2) DKK is involved in LSC maintenance independent of its role in inhibiting Wnt signaling. Together, these studies suggest that canonical Wnt signaling regulates the proliferation of human LSCs [62].

The role of non-canonical Wnt/PCP and Wnt/Ca^2+^ pathways in LSC regulation is largely unknown but may involve Fzd7. Fzd7 is found preferentially expressed in the basal layer of the limbal epithelium [55] and is capable of mediating both canonical Wnt/β-catenin and non-canonical Wnt/PCP signaling in human cancers [65,66], Xenopus foregut development [67], and rat hippocampal dendrite formation [68]. In a subset of basal limbal epithelial cells, Fzd7 colocalized with syndecan-4 and fibronectin [55]. The Fzd7/syndecan-4/fibronectin complex has been shown to induce symmetric division of muscle satellite stem cells when bound with Wnt7a [69]. One study in rabbit LSCs suggests that the Fzd7/syndecan-4/fibronectin complex may bind to Wnt11 and increase proliferation via non-canonical Rho/ROCK [70]. Knockdown of the Wnt receptor Fzd7 in cultivated LSCs results in decreased colony-forming efficiency and mRNA expression of stemness markers such as ABCG2 and ΔNp63, suggesting that Fzd7 is involved in the maintenance of the LSC stem cell phenotype [55]. These studies suggest that non-canonical signaling also plays an important role in LSC regulation and controlled canonical Wnt activation is necessary to maintain healthy LSCs. While the canonical and non-canonical Wnt pathways both appear to affect LSC proliferation and colony-forming ability, currently available data suggest that the Wnt pathways increase proliferation through distinct mechanisms. Namely, canonical Wnt causes LSC proliferation via β-catenin/TCF4/survivin pathway, while non-canonical Wnt may induce proliferation via Fzd7/syndecan-4/fibronectin/ROCK.

It is important to note that individual Wnt ligands can activate different pathways depending on their concentration [71,72,73,74]; Wnt ligands are active primarily close by to where they were secreted [75], and even Wnt ligands that typically activate non-canonical signaling can synergize to activate canonical Wnt/β-catenin signaling [76]. A balance between canonical and non-canonical Wnt signaling likely exists in LSCs, as shown by Wnt6 modulating both canonical and non-canonical Wnt signaling [22,71], and this balance is affected by LSC–niche interactions. The microRNA family miR103/107 targets NEDD9 for degradation to mediate E-cadherin localization to adherens junctions, p90RSK2 to arrest cells in the G0/G1 phase of the cell cycle, PTPRM to decrease gap junctions, and Wnt3a to increase proliferation in primary human LSCs cultivated on Collagen IV-coated plates [60]. Surprisingly, although Wnt3a is a commonly used recombinant Wnt ligand to activate the canonical Wnt pathway, Wnt3a addition decreases the ability of cultivated human LSCs to form holoclones [60]. The same study showed that miR103/107 inhibition using antagomirs leads to increased secreted Wnt3a, increased phosphorylated JNK (a non-canonical Wnt/PCP marker), decreased protein expression of YAP1, which is involved in proliferation, and decreased Sox9, a transcriptional target of the non-canonical Wnt/Ca^2+^ pathway [60]. Sox9 knockdown in passage 1 primary human limbal epithelial cells increases β-catenin and Wnt4 mRNA while decreasing GSK3-β mRNA. This suggests that Sox9 downstream of non-canonical Wnt/Ca^2+^ signaling antagonizes canonical Wnt/β-catenin [77]. Additionally, the HC-HA/PTX peptide present on the HAM promotes human LSC quiescence via activating the non-canonical Wnt/PCP and BMP signaling pathways in the limbal stromal fibroblasts that support the cultivated LSCs [78]. Therefore, the overall phenotypic outcome of the balance between canonical and non-canonical Wnt signaling on LSCs involves Wnt ligand concentration, cell–cell interactions, cell–basement membrane signaling, and cell cycle regulators.

### 2.2. Notch Signaling Regulates LSC Asymmetric Division and Stratification

The family of Notch receptors are heterodimeric transmembrane proteins, activated by direct cell–cell interaction with Delta-Serrate-Lag (DSL) type canonical ligands [79]. Through activation of target genes such as hair and enhancer of split (HES) and others, Notch signaling controls regulation of stem cell maintenance and tissue homeostasis (cell proliferation, differentiation, and survival) in diverse tissues and cell types [80,81]. Typically, Notch is activated by an immobilized ligand on a neighboring cell [82]. After activation, the Notch receptor is cleaved into the Notch intracellular domain (NICD) and Notch extracellular domain (NECD). The NICD undergoes post-translational modifications and translocates to the nucleus where it activates target gene transcription [80,83]. Conversely, the NECD is endocytosed into the ligand-expressing cell, where it is degraded [84].

Similar to Wnt, Notch signaling is also involved in cell fate maintenance of the corneal epithelium [85]. Notch ligands and receptors have been shown to be widely distributed across the epithelial layers in the cornea and limbus [14,62,86,87,88]. The sporadic presence of NICD, HES1, and HEY1 expression in limbal tissue suggests that Notch activation may occur intermittently when corneal regeneration is required [62]. Knockout of Hes1 in mice, a Notch signaling target gene, resulted in disruption of corneal development due to decreased cell proliferation and abnormal cell differentiation of LSCs [89]. Notch inhibition in LSCs increased the expression of the epithelial cell differentiation marker keratin (K)3, whereas Notch activation had an opposite effect [86]. Knockout of Notch1 in the mouse skin and corneal epithelium by tamoxifen-induced K5-cre Notch1lox/lox causes corneal hyperplasia and aberrant corneal epithelial proliferation marked by increased Ki67 staining [90].

Using small molecule inhibitors of Notch, one study highlighted the particular significance of Notch activation in LSC regulation [62]. Blocking Notch using two separate Notch inhibitors that target different aspects of the pathway resulted in an increase in LSC phenotype and a decrease in differentiated epithelial cells. This has been observed in both human and rat LSCs in vitro [91,92,93]. Activating Notch using immobilized Jag-1 ligand in cultivated human LSCs causes downregulation of the progenitor cell marker p63α, loss of asymmetric division, and decreased epithelial stratification [94]. Another study demonstrates that limbal niche cells prevent differentiation and over-proliferation of rat LSCs via inhibition of Notch signaling [95]. Furthermore, Notch signaling mechanisms contain many nuances that may shift the outcome of the signaling depending on the ligand position, modification, and the tissue or cell type. For example, soluble Jag-1 prevented TGFβ1-induced epithelial-to-mesenchymal transition in cultivated rabbit LSCs [96]. Soluble Jag-1 has been shown to be inhibitory on Notch signaling in NIH-3T3 cells [97] and 3T3-L1 preadipocyte cells [98], while immobilized Jag-1 activates Notch signaling. Moreover, the phenotypic outcome of Notch signaling also depends on crosstalk with other pathways and signaling molecules such as NF-κB and PPARγ interaction [99], YAP/TAZ [100], and Wnt [101,102,103], which have been demonstrated in other systems. While the role of Notch in LSC regulation is still a largely unknown research area, the studies reviewed here suggest that inhibiting Notch signaling promotes LSC maintenance in human LSCs.

### 2.3. Transforming Growth Factor β/Bone Morphogenic Protein (TGFβ/BMP) Signaling Counteracts Wnt Signaling

Transforming growth factor β (TGFβ) superfamily ligands, including bone morphogenic proteins (BMPs), activate canonical TGFβ signaling by binding to type II receptors in the plasma membrane, which phosphorylate and activate type I receptors [104] and subsequently leads to the phosphorylation of Smad-2/3 or Smad 1/5/8. Phosphorylated Smad-2/3 or Smad 1/5/8 bind to Smad4, and the entire complex is translocated to the nucleus and initiates the transcription of downstream genes. Early studies of human and rat corneas found that TGFβ receptors were more highly expressed in the basal limbal epithelium relative to the more superficial limbal epithelium and the central corneal epithelium [45,105,106]. Immunolocalization of TGFβ1 [107], TGFβ2 [107,108], TGFβ receptor I, and TGFβ receptor II [105] have been detected in the human limbus. BMP4 is upregulated in the human limbus relative to the cornea [54].

With the current knowledge of 12 BMPs, specific ligands were found to be upregulated differentially in human LSCs compared to those in the limbal mesenchymal cells, which are components of limbal niche cells in the stroma, in culture [63]. BMP4 and phosphorylated Smad 1/5/8 are upregulated in LSCs cultivated with limbal niche cells on 3D Matrigel compared to limbal niche cells or LSCs cultivated separately on 3D Matrigel [63]. Upon the reunion of limbal mesenchymal cells and limbal epithelial cells in culture, both Wnt signaling and BMP signaling were activated in LSCs. In the same study, inhibition of BMP signaling using noggin led to nuclear translocation of β-catenin in the LSCs, demonstrating activation of canonical Wnt/β-catenin signaling. Downstream, BMP inhibition simultaneously led to increased colony-forming efficiency and percentage of K12-expressing cells. This suggests that the canonical Wnt pathway is counteracted by BMP in limbal niche cells, and the balance between canonical Wnt and BMP results in LSC proliferation [63].

HAM, a substrate used to support the maintenance of cultivated LSCs to be transplanted as treatment for LSCD, supplies TGFβ among many other growth factors to the cultivated LSCs [109]. While the role of each individual growth factor provided by HAM has not been parsed, the cocktail of cytokines and growth factors provided by HAM cooperate to enable LSC proliferation and survival and support an anti-inflammatory microenvironment [110,111,112]. One possible mechanism of TGFβ1 provided in HAM is that TGFβ1 induces the production of MMP-9 in human LSCs [113], and MMP-9 facilitates ECM remodeling to promote epithelial outgrowth from the limbal explant [114], which might be one of its many regulatory roles in the expansion of LSCs.

While Smad-dependent TGFβ signaling may have a positive role in maintaining LSCs, it has also been demonstrated to induce epithelial–mesenchymal transition (EMT) in mouse and cultivated rabbit LSCs when TGFβ1 is supplemented to the culture [96,98]. In the rabbit LSC culture, TGFβ1-induced EMT is counteracted by Smad7 [96]. EMT was also accompanied by upregulated canonical Wnt/β-catenin signaling, loss of E-cadherin expression on the membrane, and a decrease in cell density [98]. This suggests that low levels of TGFβ or TGFβ signaling in balance with other signaling pathways and mechanotransductive cues (which will be explored further in the next section) support LSC quiescence but may cause EMT if this balance is disrupted.

### 2.4. Shh Could Promote Cell Cycle Progression and Prevents Terminal Differentiation in LSCs

Sonic hedgehog (Shh) signaling has been thoroughly characterized in embryonic development, and is involved in the proliferation and differentiation of dental epithelial cells [115], hair follicle development [116], and gastric epithelial development [117]. Shh itself is a protein that undergoes a post-translational autocatalytic cleavage into a secreted N-terminal domain and a C-terminal domain involved in intramolecular processing. Shh binds to the Patched (Ptc) family of transmembrane receptors, which then activate the transmembrane protein Smoothened (Smo). Following Ptc/Smo activation, the Gli family of transcription factors is translocated to the nucleus to transcribe Shh-dependent genes. Shh/Ptc/Smo activation likely causes epithelial cell proliferation through upregulating cyclin D1 to promote cell cycle progression [118]. Shh expression has been shown to be upregulated in the basal limbal epithelium. The Gli3 transcription factor is typically found in the naïve basal and superficial limbal epithelium [118].

The hypothesis that Shh is involved in activating LSCs to proliferate is supported in cultivated human and rabbit LSCs. Activation of Shh using Smoothened agonist (SAG) increased mRNA and protein expression of Sox9. Sox9 knockdown in passage 1 primary human limbal epithelial cells has been shown to paradoxically increase ΔNP63, ABCG2, K12, and K3 mRNA levels, while decreasing proliferating cell nuclear antigen (PCNA), K14, and K15 mRNA levels. This suggests Sox9 simultaneously represses stem cell- and terminally differentiated cell-related genes, instead favoring genes involved in the proliferation of progenitor cells such as the transient amplifying cells of the limbus [77]. Shh inhibition decreased colony-forming efficiency in cultivated rabbit LSCs [119]. The pigment epithelial growth factor-derived peptide 44-mer was shown to mediate LSC proliferation and maintenance via the Shh pathway, as 44-mer-treated rabbit LSCs had increased nuclear Gli1 and Gli3, and inhibition of Shh using HPI4 prevented 44-mer-induced LSC proliferation [119]. Therefore, Shh signaling is likely involved in the progression of LSCs to the proliferative transient amplifying state but prevents cells from becoming terminally differentiated.

## 3. Mechanotransduction via ECM Components in the Regulation of LSCs

Many types of stem cells experience mechanotransduction, which is the intracellular chemical response to external mechanical stimuli [120]. Corneal and limbal epithelial cells experience mechanical stimuli such as rubbing of the eyes, contact lens wear, and fluid dynamics from the tear film [121]. In culture, corneal epithelial cells are able to conform to the topography of the surface on which they are grown [122,123,124], and the components of the culture media can influence the ability of the corneal epithelial cells to conform to their substrate [125]. In addition, the response of LSCs to growth factors is influenced by their surrounding substrates [121,126]. The ECM on which LSCs grow has a considerable influence on the proliferative potential of stem cells [55,127,128]. LSCs react to their substrates via hemidesmosomes that anchor the LSCs to the basement membrane [129] and junctional complexes that link LSCs to other cells in their niche [22,130]. However, the concentrations of hemidesmosomes [129] and gap junctions are lower in the basal limbal epithelium than in the basal corneal epithelium, suggesting that mechanotransduction in LSCs might be different than in corneal epithelial cells [111]. This section of the review will discuss how various plasma membrane proteins expressed by the LSCs respond to biomechanical changes to transmit molecular signals into the cells, and how ECM components regulate LSCs.

### 3.1. Stiffness Affects Differentiation of LSCs through BMP and YAP Signaling

Recent data evaluating the biomechanical properties of the in vivo human LSC niche present valuable insights into the regulation of LSCs. The overall stiffness of the limbal epithelium, ECM, and basement membrane are lower than the central cornea, corneal basement membrane and Bowman’s layer, and differentiated CECs, respectively, as demonstrated by atomic force microscopy [131,132]. A high-resolution survey of the mechanical properties of the naïve human cornea acquired using Brillouin spectromicroscopy [133] demonstrated that the superficial epithelium of the limbus has a similar stiffness to the whole corneal epithelium. However, within the limbal epithelium, the middle wing layer and basal layer were significantly softer than the superficial layer. Interestingly, stiffness of the limbal stroma is not uniform, containing regions of high and low stiffness, whereas the central corneal stroma has uniform stiffness. Additionally, the region of soft basal epithelium corresponded to the location of cells expressing LSC markers such as ABCG2, CK15, nuclear β-catenin, laminin-γ3, integrin-α9, and ΔNp63 [133]. Primary human LSCs expressing high levels of ΔNp63 and ABCG2 are significantly softer than human LSCs cultivated for four weeks that express lower levels of ΔNp63 and ABCG2, as measured by AFM [132].

LSC depletion and stiffening of the limbus as a result of chemical injury causes LSCD [134], and an LSC population can be restored in these injured corneas when the stroma is softened using collagenase in culture [133] and in animal studies [135]. Human and bovine LSCs cultured on stiffer collagen gels differentiate and express higher levels of nuclear YAP and BMP4, while LSCs cultured on softer counterparts retain the progenitor cell marker phenotype and express proliferative markers such nuclear ΔNp63 and β-catenin [133,136,137]. In vivo, cytoplasmic YAP expression is upregulated in the human limbus relative to the cornea [138]. As discussed above, BMP4 activation improves LSC maintenance, so it appears that the downstream effect of BMP4 on LSCs depends on crosstalk with other signaling factors including YAP [133].

Bovine LSCs cultured on stiff HAM differentiate more than when they are cultured on softer HAM as demonstrated by increased K3+ cells in the LSCs cultured on stiff HAM [139,140]. The HAM has a variety of properties that complicate study of the effect of mechanical properties of the HAM on the LSCs in culture. In addition to its Wnt regulatory role, HAM also activates TGFβ signaling. It is important to consider that culturing explants on HAM alters the ECM organization of both the HAM and the stroma of the explants, which may be due to the stromal cells and not the cultivated epithelial cells [141]. However, as discussed above, MMP-2 and MMP-9 are expressed by cultivated LSCs and facilitate remodeling of HAM ECM to promote growth of the epithelial cell sheet from explants [114]. Therefore, while stiff collagen gels and HAM substrates increase LSC differentiation, it is important to consider the other factors these substrates present that affect LSC function separately from substrate stiffness, such as signaling mechanisms of the substrates and the remodeling effect the cells can have on the substrate itself.

### 3.2. Integrins and Cadherins in the Membrane Mediate LSC Responses to Their Environment

**Figure 2:**
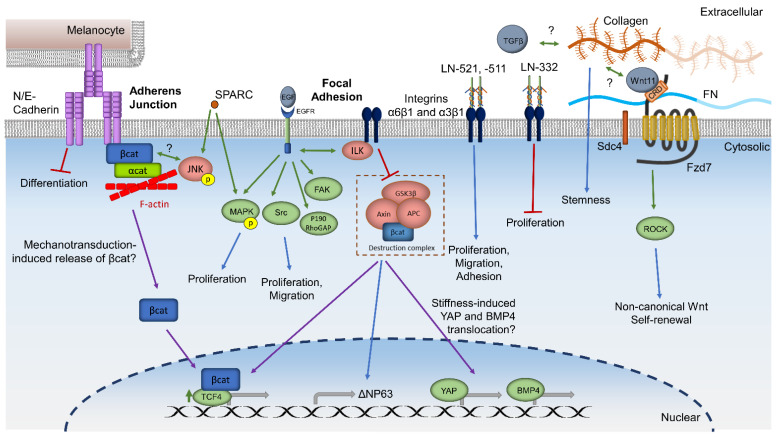
Summary and hypothesized mechanisms of ECM and membrane proteins involved in LSC regulation. LN: Laminin. FN: Fibronectin. Green arrows: activation. Red lines: inhibition. Blue arrows: downstream pathways or phenotype. Purple arrows: translocation. Gray arrows: gene expression. Double-sided arrows: crosstalk. ?: Hypothesized mechanisms based on experiments in non-corneal cells.

Integrin receptors have been found to induce cellular responses [11,12,13,55,142]. α3β1 and α6β4 integrins anchor LSCs to the limbal basement membrane in human tissue [16]. Recently Ma and colleagues have shown that Integrin-linked kinase (ILK) interacts with integrin β1 and β3 in human LSCs cultivated from limbal explants to facilitate a phosphorylation cascade. This cascade is believed to inactivate GSK3-β, thus inhibiting the degradation of β-catenin. ILK upregulates TCF-4, ΔNp63 transcription, and nuclear localization of β-catenin, indicating both an improvement in the stem/progenitor cell phenotype and activation of canonical Wnt/β-catenin signaling [143]. Cultures on collagen, laminin, or Matrigel also induced upregulation of the same factors; however, the increase was much more prominent on cross-linked epithelial HAM [143]. ILK has also been shown to mediate the balance between Wnt, TGFβ, and BMP signaling in quiescent hair follicle stem cells through ECM remodeling [144]. β1 and β3 integrins comprise focal adhesions, which mediate the cellular response to environmental cues by signaling to the cell to adjust the cytoskeleton [145]. Using immortalized human corneal keratinocytes, one study found that epidermal growth factor (EGF), a common component in LSC cultivation, activates integrin and EGF receptor crosstalk that leads to downstream activation of focal adhesion kinase, MAPK, Src, and the activated RhoA antagonist p190RhoGAP [145]. Through this mechanism, EGF signaling and integrin signaling synergize to modulate cell adhesion and improve cell motility. This connection between integrin signaling, focal adhesion signaling, and canonical Wnt signaling is supported in studies that show mechanical strain and activation of canonical Wnt signaling increase cell proliferation [132,146]. Therefore, integrin and focal adhesion signaling are a possible upstream cascade of stiffness-induced YAP nuclear localization and LSC differentiation (Figure 2). Integrin expression is also involved in Notch signaling, as Hes1 knockout mice have decreased limbal expression of integrin α6 and β1 [89]. Additional studies are necessary to confirm these signaling mechanisms in LSCs.

N-cadherin and E-cadherin are other factors mediating the tight cell–cell interactions between limbal mesenchymal cells, LSCs, and limbal melanocytes [4,16,60]. N-cadherin is only expressed in the limbus, and the expression is highest in the limbal melanocytes and the basal limbal epithelium [54,130]. N-cadherin has also been used as a marker of cultivated epithelium enriched in LSCs [55]. The association of LSCs with limbal mesenchymal cells and limbal melanocytes was shown to prevent the differentiation of LSCs through additional signaling pathways, such as Notch in rats [95]. N-cadherin may also influence Wnt signaling in LSCs as a result of mechanotransduction (Figure 2), as N-cadherin has been shown to mediate nuclear translocation of β-catenin in osteogenic differentiation resulting from fluid flow-induced mechanotransduction [44]. Studies in human keratinocytes demonstrated that β-catenin is associated with the cadherins forming adherens junctions [50]. In addition, Fzd7 was found to promote cadherin recycling to and from the membrane via Wnt11 signaling during zebrafish gastrulation [147], Xenopus development [148], and mouse cardiovascular development [149]. These studies demonstrate that N-cadherin is involved in LSC–niche interaction, which mediates downstream Notch and Wnt signaling pathways as part of the regulatory machinery.

### 3.3. The ECM Proteins Laminin, Collagen, Fibronectin, and SPARC Cooperate with Molecular Signaling Pathways to Regulate LSCs

Various laminins (LNs) are differentially expressed in the limbal basement membrane or the Bowman’s layer in the central cornea, and more recently LN-based matrices for culture of LSCs have been explored [150,151]. Coating fibrin gels with LN-521 or LN-511 (specifically the bioactive C-terminal domain of LN-511 (LN-511-E8)) increased human LSC proliferation, adhesion, and migration through integrins α6β1 and α3β1, while LN-332 on fibrin gel decreased proliferation but increased the expression of the undifferentiated marker K15 (Figure 2) [151]. Similarly, LN-521, LN-511, and LN-332 have been shown to interact with integrin α6β1, α3β1, and α6β4, respectively, in the mouse epidermis and in a biochemical assay of purified human proteins [144,152,153,154]. Together, the interaction of LNs with β1 integrins in the limbal niche could be one mechanism of upregulating Wnt/β-catenin signaling via ILK.

The epithelial basement membrane of the limbus is composed of numerous collagens [11,150], which are becoming more frequently used as a substrate in hydrogels for culturing LSCs [155,156]. Inducing ECM synthesis using ascorbic acid was shown to increase stemness markers of immortalized mouse LSCs (TKE2 cells) [157]. Importantly, this effect of ascorbic acid was primarily mediated by increased collagen production, as inhibiting collagen production decreased the expression of stemness markers (Figure 2). To address the possibility that ascorbic acid was improving the LSC phenotype through Akt signaling or antioxidant activity, mouse LSCs were treated with other antioxidants and an inhibitor of Akt signaling, but no effect on the mouse LSCs was observed in any of these conditions [158]. The stability and synthesis of collagen in the cornea may involve Wnt and TGFβ signaling (Figure 2).

Fibronectin (FN) has been shown to enhance human muscle stem cell proliferation through the non-canonical Wnt/PCP pathway by interacting with Syndecan-4 (Sdc4), a coreceptor of Frizzled-7 (Fzd7) [69,159,160]. Sdc4, Fzd7, and FN were found to colocalize in the human limbal basal epithelia [47]. Upon treatment with FN, rabbit LSCs demonstrated increased Wnt11 and Fzd7 interaction, upregulation of non-canonical Wnt/ROCK PCP ligands and receptors, and self-renewal of the LSC population (Figure 2). This same experiment also found that knocking out Wnt4 and Wnt11 in the rabbit LSCs led to differentiation of the LSCs, measured by increased expression of K3 and decreased expression of ABCG2 and ΔNP63α [70]. Importantly, stem cells may also influence their own niche. For example, activated satellite stem cells in skeletal muscle upregulate endogenous FN expression, which in turn might enhance Wnt7-Fzd7 signaling [69].

Secreted protein acidic and rich in cysteine (SPARC) is a component of the limbal basement membrane that colocalizes with ABCG2/p63-expressing LSCs in the human limbus [150]. When soluble SPARC was added to cultivated rabbit LSCs, expression of p63α and ABCG2 was found to be increased, while the differentiation marker K3 was decreased. This study also found that SPARC led to phosphorylation and activation of JNK and p38 MAPK [161]. Because inhibition of JNK leads to the formation of adherens junctions in human foreskin keratinocytes [162], SPARC may result in the dissolution of adherens junctions via JNK activation, and progression through mitosis via MAPK activation (Figure 2). However, this potential mechanism would need to be investigated in human LSCs.

## 4. Conclusions and Future Directions

LSCs are regulated by carefully tuned balances of various signaling pathways, and the activation or inhibition of these pathways is frequently dependent on the interactions of LSCs with various niche cell types and extracellular substrates. In addition to receiving molecular signals from growth factors, cytokines, and other soluble molecules, LSCs also respond to their surrounding physical structure via mechanotransduction, interaction with the ECM, and interactions with other cell types. In the presence of sufficient cell-to-cell contact, a balance among the signaling pathways including Wnt, Notch, BMP and Shh, a soft external environment and ECM, the LSCs are capable of self-renewal. When such balance is lost, the LSCs are prompted to proliferate via either symmetrical or asymmetrical division. When the rate of proliferation cannot sustain the rate of turnover from external insults or the alternation of the niche physical environment impairs the survival of LSCs, depletion of LSCs occurs as seen in LSCD.

Future study of LSC regulation should incorporate an understanding LSCs’ responses to their external cues. Small molecules that target specific signaling pathways in LSCs could be made more efficacious in combination with bioengineering approaches incorporating 3D printing, hydrogels, or matrix biology. These could be used to improve the clinical outcome of LSC transplantation, or as a topical treatment that would obviate the need for transplantation altogether. Ultimately, future development of LSCD therapies should take into consideration all of these niche factors to achieve a better long-term success.

## Data Availability

Not applicable.
